# Performance of and Factors Associated With Tuberculosis Screening and Diagnosis Among People Living With HIV: Analysis of 2012–2016 Routine HIV Data in Tanzania

**DOI:** 10.3389/fpubh.2019.00404

**Published:** 2020-02-06

**Authors:** Werner Maokola, Bernard Ngowi, Lovetti Lawson, Michael Mahande, Jim Todd, Sia E. Msuya

**Affiliations:** ^1^National AIDS Control Program/Ministry of Health, Community Development, Gender, Elderly and Children, Dar es Salaam, Tanzania; ^2^Institute of Public Health, Kilimanjaro Christian Medical University College, Moshi Urban, Tanzania; ^3^National Institute of Medical Research, Dar es Salaam, Tanzania; ^4^Zanklin Medical Center, Abuja, Nigeria; ^5^London School of Hygiene and Tropical Medicine, London, United Kingdom

**Keywords:** tuberculosis, screening, diagnosis, HIV, Tanzania

## Abstract

People Living with HIV (PLHIV) should be screened for tuberculosis (TB) at every visit to the HIV care and treatment clinic (CTC), and those with positive results on screening should undergo further diagnostic investigations. We evaluated the performance of the TB diagnosis cascade among PLHIV attending CTC between January 2012 and December 2016 in three regions of Tanzania: Dar es Salaam, Iringa, and Njombe. We used descriptive epidemiology to evaluate performance and logistic regression to determine odds ratios (OR) for factors associated with TB screening and further TB diagnosis after positive TB screening. We analyzed 169,741 PLHIV who made 2,638,876 visits to CTC between January 2012 and December 2016. We excluded 2,074 (0.80%) visits as these involved PLHIV enrolled in CTC with a prior TB disease diagnosis. Of the 2,636,802 visits, 2,524,494 (95.67%) had TB screening according to national guidelines, of which 88,028 (3.49%) had TB screening positive results. Of the 88,028 visits with a positive TB screening, 27,810 (31.59%) had no records for further TB diagnosis following positive TB screening. Of all visits with positive TB screening, 32,986 (37.50%) had a TB disease diagnosis. On multivariate logistic regression, those who visited with World Health Organization (WHO) clinical stage four (aOR = 3.61, 95% CI 3.48–3.75, *P* < 0.001), enrolled in health center (aOR = 1.26, 95% CI 1.24–1.29, *P* < 0.001), enrolled in Iringa region (aOR = 1.54, 95% CI 1.50–1.57, *P* < 0.001), and enrolled in 2015 (aOR = 1.20, 95% CI 1.18–1.24, *P* < 0.001) were more likely to have no TB screening. Visits involving those who were of the female sex (aOR = 1.14, 95% CI 1.11–1.18, *P* < 0.001), enrolled in Njombe region (aOR = 4.36, 95% CI 4.09–4.65, *P* < 0.001), and enrolled in 2016 (aOR = 2.62, 95% CI 2.49–2.77, *P* < 0.001) were more likely to have no further TB diagnosis after positive TB screening. The study documented high performance of TB screening for PLHIV in HIV CTCs but a low transition of presumptive TB case undergoing further investigations. Better systems are needed for ensuring presumptive TB cases are diagnosed including using more efficient diagnostic methods like Gene pert.

## Introduction

Despite wide use of antiretroviral (ARV) drugs, tuberculosis (TB) is still a public health challenge for People Living with HIV (PLHIV) (1). In autopsy studies among PLHIV in Africa, TB was present in 21–54% of people, and TB was the cause of death in 32–45% of the PLHIV in the study (2). The risk of TB is higher among PLHIV; HIV-positive individuals are up to 26 times more likely to have active TB disease compared to HIV negative individuals, and globally at least 30% of PLHIV have latent TB (3). Worldwide, in 2018, 10.0 million TB cases were notified, and of these 24% were from the African continent. TBHIV accounted for 8.6% of the notified TB cases. Of the 1.5 million TB deaths reported in 2018, 17% (251,000) of deaths were HIV positive (1). In Tanzania, which is among 30 high TB-burdened countries, a total of 142,000 TB cases were diagnosed with TB disease in 2018, of which 40,000 (28%) were HIV positive (1).

To reduce TB among PLHIV, the World Health Organization (WHO) recommends Intensified TB case finding (ICF) among PLHIV attending HIV care and treatment clinics (CTC), which entails active TB screening for symptoms and signs using standardized TB screening questions (4). In Tanzania, those who screen positive undergo further TB diagnosis according to the National Guidelines (5). TB screening using symptoms and signs among PLHIV is important to increase TB disease diagnosis, and ICF is thus a gateway to TB management among PLHIV (6, 7). There is an urgency to build better TB prevention in resource-limited settings, but to do so we need to evaluate how well the current cascade is working (8).

TB diagnosis cascade among PLHIV in CTCs faces a number of challenges, including those lost to follow up in the diagnosis cascade (9, 10). A study in Northern Uganda looking at improved TB case notification among both HIV-positive and HIV-negative individuals found a TB positivity rate of 3.5% among the 385 HIV positive who were positive on the screening questions. A prospective study in India found that 30% screened TB positive, and 35% of these were referred for TB diagnostic tests, and 15% had confirmed TB (11). In Kenya, a study reported a high TB screening among 1,020 newly diagnosed PLHIV; 98% of PLHIV were screened, but only 16% of those screened positive underwent further TB diagnosis evaluation, and 26 (2.6%) were eventually diagnosed with TB (12). In Ethiopia, 72% of PLHIV with positive TB screening were linked to sputum microscopy for TB with the remaining PLHIV diagnosed using other methods (13). A study conducted in Ghana also found that sputum for smear microscopy was requested for 58.7% of those who needed (14).

In this paper, we analyzed routine HIV data from PLHIV enrolled in CTC from January 2012 and December 2016. We evaluated the performance of the TB diagnosis cascade for PLHIV attending CTC and determined factors associated with TB screening and further TB diagnosis after positive TB screening. The aim of the paper was to provide evidence to the Tanzanian Ministry of Health Community Development, Gender, Elderly and Children (MoHCDGE) on the performance of TB diagnosis cascade among PLHIV attending CTC in Tanzania for quality improvement. The findings from this study will also be applicable to other developing countries where HIV and TB are also prevalent.

## Materials and Methods

The study involved electronic patients' records that were routinely collected and contained in the CTC database from 317 health facilities in three regions: Iringa, Njombe, and Dar-es-Salaam. The study used retrospective data from PLHIV enrolled in CTC from January 2012 to December 2016. PLHIV enrolled into CTC with an existing diagnosis of TB disease from TB clinics were excluded from the study.

The United Republic of Tanzania comprises of mainland Tanzania and Zanzibar with a population of about 44 million in 2012 (15). Tanzania Mainland has a generalized HIV epidemic with the first case of HIV reported in 1983. By 1986, all regions in the country had reported at least one case (16). The Tanzania HIV Indicator Survey showed an overall prevalence of HIV of 5.1% in 2011/2012 and 4.7% in 2016/17. The three regions were purposely selected as they had higher HIV prevalence according to the recent National HIV survey (17).

**Figure 1:**
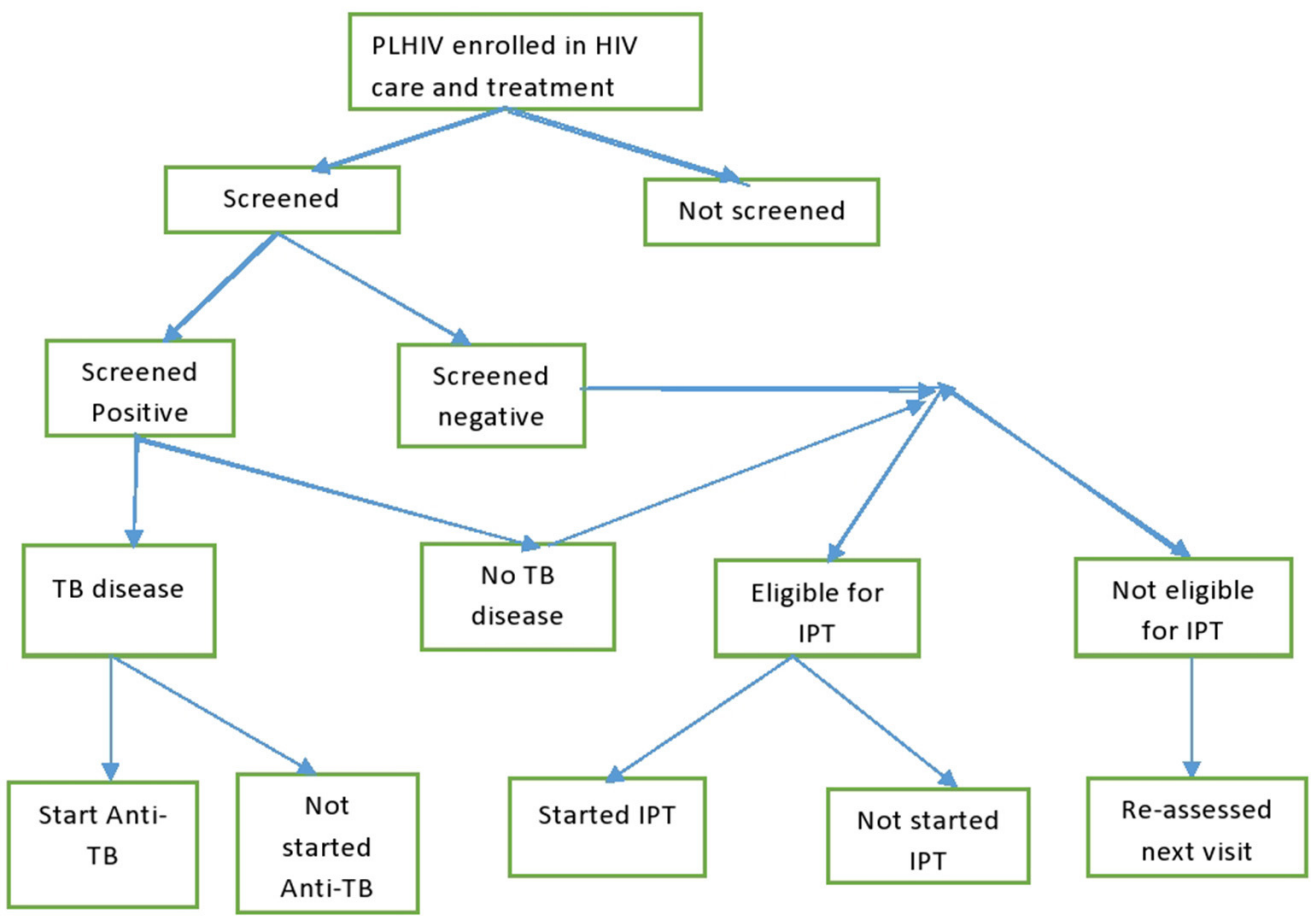
TB diagnosis cascade in HIV care and treatment clinics.

Tanzania have rolled out widespread, freely available antiretroviral therapy services in HIV CTC ever since 2004. For TB management in CTC, PLHIV were screened for TB using the standardized WHO questions at every visit to the clinics. Those who screened positive were further evaluated for TB disease using either sputum examination, radiology, or clinical diagnosis according to a TB management algorithm (5). Those with confirmed TB disease were started on anti-TB therapy. PLHIV who were negative on the TB screening were assessed for their eligibility for Isoniazid Preventive Therapy (IPT). Those who became eligible for IPT were kept on daily Isoniazid tablets for 6 months to prevent active TB disease. TB infection control measures were another set of interventions implemented in HIV clinics to prevent TB transmission among PLHIV and staff (Figure 1).

### Data Extraction Process and Variables Descriptions

De-identified secondary data were extracted from the main national HIV care and treatment database. The independent variables of interest included age, sex, WHO clinical stage, health facility type (Hospital, Health center or Dispensary), health facility ownership (public or private/Faith Based Organization), region during CTC enrolment, TB screening status (screen or not screened), and TB diagnosis status after positive TB screening (Further TB diagnosis or no further TB diagnosis). The final dataset excluded all PLHIV with known TB diagnosis before CTC enrolment.

### Data Analysis

Stata version 14 (Stata Corporation, College Station, Texas, USA) was used for data cleaning, merging, and analysis. Descriptive statistics used mean and standard deviation for continuous variables and frequencies and proportions for categorical variables. For each estimate, a 95% Confidence Interval (95% CI) was also calculated. Bivariate logistic regression was used to determine odds ratios (OR), and 95% CI for the association between independent and dependent variables. The likelihood ratio test was used to obtain chi-square statistic and *p*-value for each analysis. Finally, all independent variables with a *p* of ≤0.2 in a bivariate analysis were subjected in a multivariate logistic regression to determine the adjusted OR and 95% CI for independent factors associated with TB screening and further TB diagnosis after positive TB screening. In the final analysis, a cut-off *p* ≤ 0.05 was considered statistically significant.

### Ethical Consideration

The study involved secondary analysis of unlinked data; hence, there was no contact with human subjects. The permission to conduct the study was obtained from the National Institute of Medical Research-Tanzania, and permission to use routinely collected data was obtained from National AIDS Control Program by signing a data transfer agreement. This study was carried out as part of the PhD research by Werner Maokola with ethical permission for the PhD from KCMU college.

## Results

A total of 171,743 PLHIV were enrolled in HIV CTC in the Dar es Salaam, Iringa, and Njombe regions from January 2012 to December 2016. The mean age for the PLHIV was 35 years (Range: 0–97 years). A total of 2,074 PLHIV were not included in the analysis as they had TB disease before CTC enrolment. Of the remaining 171,669, the majority of PLHIV were aged 25–49 years; 123,581 (71.99%), females; 117,925 (68.69%), had working functional status; 164,076 (95.58%), and had WHO clinical stage one; 62,535 (36.43%). Most of the study participants were also enrolled in 2016, 37,788 (22.01%), were from dispensary level, 65,405 (38.10%), and were from public health facilities, 123,964 (72.2%) (Table 1).

**Table 1:** Baseline characteristics of PLHIV enrolled in CTC between 2012 and 2016 in three regions of Tanzania *N* = 171,669.

**Variable**		**Total, *N***	**Dar-es-Salaam, *N***	**Iringa, *N***	**Njombe, *N***
**Individual level factors**					
**Age (years)**	**Mean = 35 years**, ***SD*** **= 12 years, Range = 0–97 years**				
	<1	1,361 (0.79%)	946 (0.77%)	183 (0.92%)	232 (0.81%)
	1–4	3,497 (2.04%)	2,144 (1.74%)	569 (2.88%)	784 (2.75%)
	5–9	2,84 (1.65%)	1,707 (1.38%)	497 (2.51%)	637 (2.23%)
	10–19	7,564 (4.41%)	5,103 (4.14%)	884 (4.47%)	1,577 (5.52%)
	20–24	16,453 (9.58%)	11,781 (9.55%)	1,66 (8.42%)	3,006 (10.53%)
	25–49	123,581 (71.99%)	89,722 (72.75%)	13,998 (70.73%)	19,861 (69.57%)
	>50	16,363 (9.53%)	11,92 (9.67%)	1,990 (10.06%)	2,452 (8.59%)
	Missing	9 (0.01%)	5 (0.00%)	3 (0.02%)	1 (0.00%)
**Sex**	Male	53,744 (31.31%)	34,657 (28.10%)	8,034 (40.59%)	11,054 (38.72%)
	Female	117,9275 (68.69%)	88,672 (71.90%)	11,757 (59.41%)	17,496 (61.28%)
**Functional status**	Ambulatory	5,426 (3.16%)	3,634 (2.95%)	1,078 (5.45%)	713 (2.50%)
	Bedridden	1,419 (0.83%)	1,061 (0.86%)	181 (0.91%)	177 (0.62%)
	Working	164,076 (95.58%)	118,038 (95.71%)	18,478 (93.37%)	27,560 (96.53%)
	Missing	749 (0.44%)	596 (0.48%)	53 (0.27%)	100 (0.35%)
**WHO stage**	Stage 1	62,535 (36.43%)	48,400 (39.24%)	5,140 (25.97%)	8,995 (31.51%)
	Stage 2	40,347 (23.50%)	26,587 (21.56%)	5,707 (28.84%)	8,053 (28.21%)
	Stage 3	53,846 (31.37%)	38,648 (31.34%)	7,062 (35.68%)	8,136 (28.50%)
	Stage 4	12,748 (7.43%)	8,078 (6.55%)	1,580 (7.98%)	3,090 (10.82%)
	Missing	2,193 (1.28%)	1,616 (1.31%)	301 (1.52%)	276 (0.97%)
**Enrolment year**	2012	31,038 (18.08%)	20,359 (16.51%)	5,083 (25.68%)	5,596 (19.60%)
	2013	33,403 (19.46%)	24,230 (19.65%)	3,746 (18.93%)	5,427 (19.01%)
	2014	36,091 (21.02%)	26,232 (21.27%)	3,718 (18.79%)	6,141 (21.51%)
	2015	33,349 (19.43%)	24,433 (19.81%)	3,566 (17.97%)	5,360 (18.77%)
	2016	37,788 (22.01%)	28,075 (22.76%)	3,687 (18.63%)	6,026 (21.11%)
**Type of health facility**	Dispensary	65,405 (38.10%)	52,236 (42.35%)	7,409 (37.44%)	5,760 (20.18%)
	Health center	45,659 (26.60%)	27,910 (22.63%)	8,633 (43.62%)	9,117 (31.93%)
	Hospital	60,606 (35.30%)	43,183 (35.01%)	3,749 (18.94%)	13,673 (47.89%)
**Health Facility ownership**	Private	47,705 (27.79%)	30,962 (25.11%)	10,149 (51.28%)	6,594 (23.10%)
	Public	123,964 (72.21%)	92,367 (74.89%)	9,641 (48.72%)	21,956 (79.90%)

*WHO, World Health Organization*.

**Figure 2:**
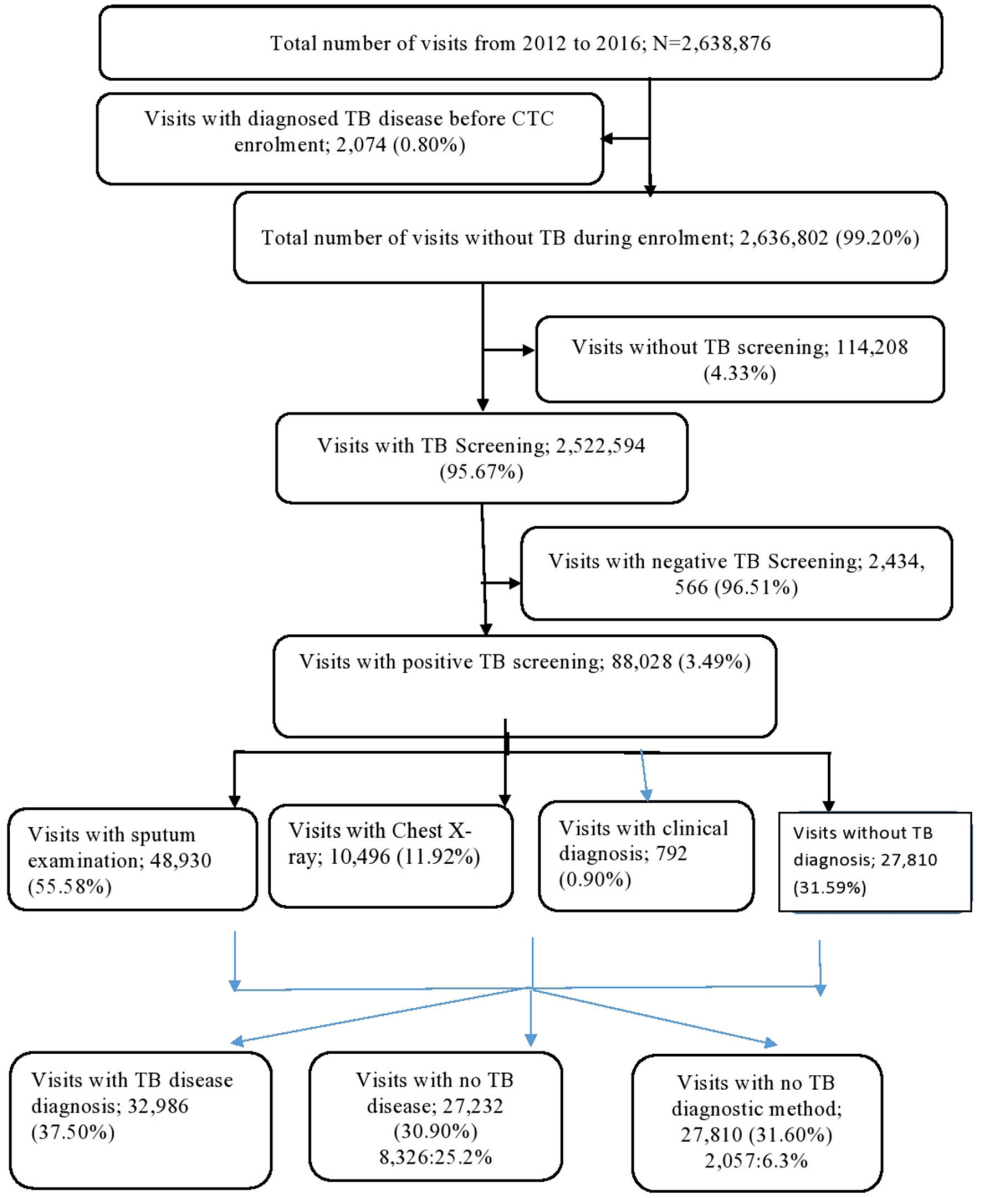
TB diagnosis cascade for PLHIV in HIV CTCs.

The study cohort made a total of 2,638,876 visits during the follow-up period; however, 2,074 visits (0.80%) visits were excluded from the analysis as they involved TB disease diagnosis before CTC enrolment. Of the remaining 2,636,802 visits, 2,524,494 (95.67%) had TB screening. Of these, 88,028 (3.49%) had TB-positive screening results. Of the visits with TB screening, 48,930 (55.58%) had sputum examinations, 10,496 (11.92%) had a chest X-ray, 792 (0.90%) had a TB diagnosis through clinical criteria, and 27,810 had no records for further TB diagnosis following positive TB screening. Of all visitors with a positive TB screening, 32,986 (37.50%) had a TB disease diagnosis (Figure 2).

### Factors Associated With TB Screening

On multivariate logistic regression analysis, visitors with WHO clinical stage four (aOR = 3.61, 95% CI 3.48–3.75, *P* < 0.001) and who were enrolled in a health center (aOR = 1.26, 95% CI 1.24–1.29, *P* < 0.001), enrolled in the Iringa region (aOR = 1.54, 95% CI 1.50–1.57, *P* < 0.001), and enrolled in 2015 (aOR = 1.20, 95% CI 1.18–1.24, *P* < 0.001) were more likely to have no TB screening. Visits that involved females (aOR = 0.68, 95% CI 0.67–0.69, *P* < 0.001) and working functional status (aOR = 0.45, 95% CI 0.43–0.48, *P* < 0.001) were less likely to have no TB screening (Table 2).

**Table 2:** Factors associated with TB screening: *N* = 2,636,691.

**Variable**	**Number not screened (%)**	**Crude**		**Adjusted**	
		**OR (95% CI)**	***P*-value**	**aOR(95% CI)**	***P*-value**
**Age group (years)**					
<1	241 (0.21%)	1			
1–4	3,416 (2.99%)	1.53 (1.34–1.75)	< 0.001	0.99 (0.82–1.19)	0.815
5–9	4,506 (3.95%)	1.87 (1.64–2.13)	< 0.001	0.95 (0.79–1.14)	0.600
10–19	5,624 (4.92%)	1.33 (1.17–1.52)	< 0.001	0.95 (0.79–1.14)	0.581
20–24	4,704 (4.12%)	0.73 (0.64–0.83)	< 0.001	0.95 (0.79–1.14)	0.565
25–49	80,692 (70.66%)	1.04 (0.91–1.18)	0.543	1.04 (0.88–1.25)	0.597
+50	15,022 (13.15%)	1.24 (1.09–1.41)	0.001	0.99 (0.83–1.19)	0.955
**Sex**					
Male	47,466 (41.56%)	1		1	
Female	66,742 (58.44%)	0.60 (0.59–0.61)	< 0.001	0.68 (0.67–0.69)	< 0.001
**Functional status**					
Ambulatory	1,630 (2.94%)	1		1	
Bed-ridden	372 (0.67%)	0.57 (0.50–0.62)	< 0.001	0.75 (0.66–0.85)	< 0.001
Working	53.381 (96.39%)	0.25 (024–0.27)	< 0.001	0.45 (0.43–0.48)	< 0.001
**Who clinical stage**					
Stage one	9,240 (14.01%)	1		1	
Stage two	11,128 (16.87%)	1.48 (1.44–1.52)	< 0.001	2.01 (1.94–2.08)	< 0.001
Stage three	37,751 (57.23%)	2.50 (2.45–2.56)	< 0.001	3.33 (3.23–3.42)	< 0.001
Stage four	7,843 (11.89%)	2.49 (2.42–2.57)	< 0.001	3.61 (3.48–3.75)	< 0.001
**Health facility type**					
Dispensary	42,902 (37.56%)	1		1	
Health center	35,043 (30.68%)	1.08 (1.06–1.09)	< 0.001	1.26 (1.24–1.29)	< 0.001
Hospital	36,263 (31.75%)	0.94 (0.93–0.96)	< 0.001	0.94 (0.92–0.96)	< 0.001
**Health facility ownership**					
Private	24,698 (21.63%)	1		1	
Public	89,510 (78.37%)	1.59 (1.56–1.61)	< 0.001	2.39 (2.34–2.45)	< 0.001
**Region**					
Dar es Salaam	73,363 (64.24%)	1		1	
Iringa	16,386 (14.35%)	1.17 (1.15–1.19)	< 0.001	1.54 (1.50–1.57)	< 0.001
Njombe	24,459 (21.42%)	1.15 (1.13–1.16)	< 0.001	0.21 (0.21–0.22)	< 0.001
**Enrolment year**					
2012	23,886 (20.19%)	1		1	
2013	24,589 (21.53%)	1.07 (1.05–1.09)	< 0.001	1.01 (0.99–1.04)	0.275
2014	24,673 (21.60%)	1.19 (1.17–1.21)	0.001	1.07 (1.04–1.09)	< 0.001
2015	21,177 (18.54%)	1.44 (1.42–1.47)	< 0.001	1.20 (1.18–1.24)	< 0.001
2016	19,883 (17.41%)	1.84 (1.80–1.87)	0.001	1.15 (1.12–1.19)	0.001

### Factors Associated With Further TB Diagnosis After Positive TB Screening

Upon multivariate logistic regression analyses, visits involving the female sex (aOR = 1.14, 95% CI 1.11–1.18, *P* < 0.001), enrolment in the Njombe region (aOR = 4.36, 95% CI 4.09–4.65, *P* < 0.001), and enrolment in 2016 (aOR = 2.62, 95% CI 2.49–2.77, *P* < 0.001) were more likely to have no further TB diagnosis after a positive TB screening. Visitors with working functional status (aOR = 0.62, 95% CI 0.58–0.67, *P* < 0.001), enrolment in hospitals (aOR = 0.39, 95% CI 0.38–0.41, *P* < 0.001), and attending public health facilities (aOR = 0.68, 95% CI 0.65–0.71) were less likely to have no further TB diagnosis after positive TB screening (Table 3).

**Table 3:** Factors associated with no further TB diagnosis among screened positive: *N* = 88,008.

**Variable**	**Number of visits screened TB positive**	**Number of visits without further TB diagnosis (%)**	**Crude**		**Adjusted**	
			**OR(95% CI)**	***P*-value**	**aOR(95% CI)**	***P*-value**
**Age group (years)**						
<1	352	137 (0.49%)	1		1	
1–4	2,395	799 (2.87%)	0.79 (0.62–0.99)	0.040	0.90 (0.69–1.17)	0.431
5–9	2,307	954 (3.43%)	1.11 (0.88–1.39)	0.388	1.11 (0.86–1.44)	0.434
10–19	3,811	1,456 (5.24%)	0.97 (0.78–1.21)	0.792	0.98 (0.76–1.26)	0.872
20–24	3,293	1,299 (4.67%)	1.02 (0.82–1.28)	0.848	0.89 (0.69–1.15)	0.376
25–49	63,911	19,533 (70.24%)	0.69 (0.56–0.86)	0.001	0.68 (0.54–0.87)	0.002
50+	11,939	3,631 (13.06%)	0.69 (0.55–0.85)	0.001	0.71 (0.56–0.91)	0.007
**Sex**						
Male	37,345	11,230 (40.38%)	1			
Female	50,683	16,580 (59.62%)	1.13(1.10–1.16)	< 0.001	1.14 (1.11–1.18)	< 0.001
**Functional status**						
Ambulatory		1,701 (6.12%)	1		1	
Bed-ridden		273 (0.98%)	0.92 (0.79–1.07)	0.305	0.81 (0.69–0.96)	0.013
Working		25,799 (92.89%)	0.94 (0.88–0.99)	0.028	0.62 (0.58–0.67)	< 0.001
**Who clinical stage**						
Stage one	5,203	2,682 (9.71%)	1		1	
Stage two	10,895	5,714 (20.68%)	1.04 (0.97–1.11)	0.286	1.04 (0.97–1.11)	0.307
Stage three	56,865	15,427 (55.84%)	0.35 (0.33–0.37)	< 0.001	0.36 (0.34–0.39)	< 0.001
Stage four	12,021	3,806 (13.78%)	0.43 (0.41–0.47)	< 0.001	0.46 (0.43–0.49)	< 0.001
**Health facility type**						
Dispensary	23,195	10,108 (36.35%)	1		1	
Health center	19,257	7,192 (25.86%)	0.77 (0.74–0.80)	< 0.001	0.78 (0.75–0.82)	< 0.001
Hospital	45,576	10,510 (37.79%)	0.39 (0.38–0.40)	< 0.001	0.39 (0.38–0.41)	< 0.001
**Health facility ownership**						
Private	16,527	6,561 (23.59%)	1		1	
Public	71,501	21,249 (76.41%)	0.64 (0.62–0.67)	< 0.001	0.68 (0.65–0.71)	< 0.001
**Region**						
Dar es Salaam	73,896	22,133 (79.59%)	1		1	
Iringa	8,823	2,179 (7.84%)	0.77 (0.73–0.80)	< 0.001	0.42 (0.39–0.45)	< 0.001
Njombe	5,309	3,498 (12.58%)	4.52 (4.26–4.79)	< 0.001	4.36 (4.09–4.65)	< 0.001
**Enrolment year**						
2012	24,503	5,898 (21.21%)	1		1	
2013	24,474	6,246 (22.46%)	1.08 (1.04–1.13)	< 0.001	1.09(1.04–1.14)	< 0.001
2014	16,923	5,973 (21.48%)	1.72 (1.65–1.80)	< 0.001	1.71 (1.63–1.79)	< 0.001
2015	12,251	5,040 (18.12%)	2.20 (2.10–2.31)	< 0.001	2.17 (2.06–2.28)	< 0.001
2016	9,877	4,653 (16.73%)	2.81 (2.67–2.95)	< 0.001	2.62 (2.49–2.77)	< 0.001

## Discussion

The present study provided an analysis of the TB diagnosis cascade for PLHIV enrolled in HIV CTCs in three regions with high HIV prevalence in Tanzania. The study cohort consisted mainly of all PLHIV attending CTC with majority at early stages of the infection (WHO clinical stage one and working functional status). Efforts have been made to make sure that people know their HIV status and that those found to be infected enroll into care. TB is the most common comorbidity among PLHIV, and it is important to ensure all those enrolled in CTC are assessed, diagnosed, and treated at every opportunity in accordance with Tanzania national Guidelines.

Ninety-five percent (95.67%) of the visitors recorded in the study cohort had TB screening. Routine TB screening is in line with the WHO recommendations (18), and such high TB screening scores among PLHIV has also been reported in other studies (12, 19–21). TB screening is known to be good to rule out TB disease in PLHIV with a negative predictive value of 97.7% (22). It is important to know that TB screening is consistently carried out in all CTC, but we found that TB screening was unlikely among PLHIV with advanced disease (WHO clinical stage 4), enrolment in health centers, enrolment in the Iringa region, and who enrolled in 2015. Health care providers may find it easier to administer TB screening questionnaire to healthier PLHIV than to those with unfavorable functional status who may already have had other investigations for comorbidities. Our observation that TB screening performance varied across health facility types, across administrative regions, and across implementation time calls for tailored interventions to improve TB screening.

Our study found that only 3.49% of visits resulted in a positive screening score over a 4 year period. This was similar to a study in Kenya where routine screening in a cohort of PLHIV attending HIV services had <1% visits where a positive screen was recorded (21). In another study in Kenya among 1,060 newly enrolled PLHIV, 62% reported symptoms of TB, but only 26 cases of TB were found. This demonstrates the higher prevalence of symptoms among PLHIV prior to attending HIV services and the low sensitivity of TB screening (12). Our finding is similar to other longitudinal repeated screening of cohorts of PLHIV (21). The lower positive TB screening in cohorts attending HIV services can be attributed to several factors. In these programs, ART is now available to all PLHIV, and this is known to reduce TB morbidity, mortality and incidence (8). Moreover, in 2014, isoniazid prevention therapy (IPT) was introduced in Tanzania for PLHIV attending CTC, with a decline in TB incidence over the 6 years of follow up (23). Conversely, some PLHIV may not have typical symptoms of TB disease and may be missed by the screening, although screening is reported to have a 97% NPV for ruling out TB infection in PLHIV (24). Further analysis of whether low positive TB screening was a reality or due to a weaknesses in the screening process will be evaluated in subsequent analyses.

Up to 31.59% of visits did not receive a further TB diagnosis procedure after positive TB screening. In our study, we found that lack of further follow up after a positive TB screening was higher among females, those enrolled in Njombe region, and those enrolled in 2016. Studies in Tajikistan and Uganda reported a loss to follow up in TB diagnosis of 15 and 33.4%, respectively. A case-control study in Tajikistan documented risk factors for loss of follow up in TB diagnosis: movements, drug side effects, previous TB treatment, patient refusal, stigma, and family problems (25). A retrospective cohort study of 646 records of PLHIV initiated on ART in Uganda found that loss to follow up was associated with good health (normal weight), attendance in Hospitals, and having no telephone contact (26). Resources to train health care workers have been set aside to make sure that PLHIV are screened at every clinic visit in Tanzania, but further resources are needed to ensure the next step in the TB cascade is taken, and further investigations are undertaken on all who screen positive.

The study found low use of sputum examination for TB diagnosis (55.58%). This finding is lower than those reported in Ethiopia and another study in Tanzania. In Ethiopia, the AFB diagnostic method was up to 72% of all the TB diagnostic methods (13), whereas in Tanzania, AFB microscopy was reported to be the most available TB diagnostic method (27). Generally, our study found lower use of sputum microscopy examination for TB disease diagnosis unlike in Ethiopia and Tanzania (13, 27). In Tanzania, a sputum examination is the first test for TB disease diagnosis. All people presumed to have TB disease have to undergo a sputum examination. Other diagnostic tests such as chest radiography and clinical diagnosis come later in the algorithm (5).

Our study found only 37.50% of visitors among positive TB screening had TB disease. Such a high TB diseases diagnosis among PLHIV with positive TB screening results was recorded elsewhere in Kenya (21). It is important to note that TB screening tools with high specificity for TB symptoms are important in TB control as resources for TB diagnosis and will be used efficiently for those who most likely have TB disease.

## Conclusion

The study documented the high performance of TB screening for PLHIV in HIV CTCs, but a low transition of suspected TB cases undergoing further investigations. Better systems are needed to ensure suspected TB cases are diagnosed, and this includes using more efficient diagnostic methods like GeneXpert.

## Data Availability Statement

The datasets generated for this study are available on request to the corresponding author.

## Author Contributions

WM conceptualized the idea of the study, designed the study, analyzed data, interpreted the results, and drafted the manuscript. MM and JT helped to analyze the data and interpret the results. BN, LL, MM, JT, and SM reviewed the manuscript. All authors approved the final version of the manuscript.

### Conflict of Interest

The authors declare that the research was conducted in the absence of any commercial or financial relationships that could be construed as a potential conflict of interest.
